# The Metabolic Profile of Plasma During Epileptogenesis in a Rat Model of Lithium–Pilocarpine-Induced Temporal Lobe Epilepsy

**DOI:** 10.1007/s12035-025-04719-6

**Published:** 2025-02-04

**Authors:** Fatma Merve Antmen, Emir Matpan, Ekin Dongel Dayanc, Eylem Ozge Savas, Yunus Eken, Dilan Acar, Alara Ak, Begum Ozefe, Damla Sakar, Ufuk Canozer, Sehla Nurefsan Sancak, Ozkan Ozdemir, Osman Ugur Sezerman, Ahmet Tarık Baykal, Mustafa Serteser, Guldal Suyen

**Affiliations:** 1 Department of Physiology, Institute of Health Sciences, Acibadem Mehmet Ali Aydinlar University, Istanbul, Türkiye; 2 Acibadem Mehmet Ali Aydinlar University, Biobank Unit, Istanbul, Türkiye; 3 School of Medicine, Department of Medical Biochemistry, Acibadem Mehmet Ali Aydinlar University, Istanbul, Türkiye; 4 Medical Laboratory Techniques, Vocational School of Health Services, Acibadem Mehmet Ali Aydinlar University, Istanbul, Türkiye; 5 Faculty of Arts and Sciences, Department of Molecular Biology and Genetics, Acibadem Mehmet Ali Aydinlar University, Istanbul, Türkiye; 6 Department of Molecular Biology and Genetics, Inonu University, Malatya, Türkiye; 7 School of Medicine, Acibadem Mehmet Ali Aydinlar University, Istanbul, Türkiye; 8 School of Medicine, Department of Basic Medical Sciences, Medical Biology, Acibadem Mehmet Ali Aydinlar University, Istanbul, Türkiye; 9 School of Medicine, Department of Basic Medical Sciences, Biostatistics and Medical Informatics, Acibadem Mehmet Ali Aydinlar University, Istanbul, Türkiye; 10 Acibadem Labmed Clinical Laboratories, Istanbul, Türkiye; 11 School of Medicine, Department of Physiology, Acibadem Mehmet Ali Aydinlar University, Istanbul, Türkiye

**Keywords:** Epileptogenesis, NMR, Epilepsy, Metabolomics, Plasma, Rat

## Abstract

Temporal lobe epilepsy (TLE) arises mostly because of an initial injury. Certain stimuli can make a normal brain prone to repeated, spontaneous seizures via a process called epileptogenesis. This study examined the plasma metabolomics profile in rats with the induced TLE to identify feasible biomarkers that can distinguish progression of epileptogenesis in three different time points and reveal the underlying mechanisms of epileptogenesis. Status epilepticus (SE) was induced by repetitive intraperitoneal injections of low-dose lithium chloride–pilocarpine hydrocholoride. Blood samples were collected 48 h, 1 week, and 6 weeks after SE, respectively. Plasma metabolites were analyzed by nuclear magnetic resonance (NMR) spectrometry. Statistical analysis was performed using MetaboAnalyst 6.0. An orthogonal partial least squares discriminant analysis (OPLS-DA) model was employed to represent variations between the TLE model groups and respective controls. Volcano plot analysis was used to identify key features, applying a fold-change criterion of 1.5 and a *t*-test threshold of 0.05. 48 h after SE, dimethyl sulfone (DMSO_2_) and creatinine levels were decreased, whereas glycine and creatine levels were increased. The only metabolite that changed 1 week after SE was pyruvic acid, which was increased compared to its control level. Lactic acid, pyruvic acid, and succinic acid levels were increased 6 weeks after SE. The identified metabolites were especially related to the tricarboxylic acid cycle and glycine, serine, and threonine metabolism. The results illustrate that distinct plasma metabolites can function as phase-specific biomarkers in TLE and reveal new insights into the mechanisms underlying SE.

**Supplementary Information:**

The online version contains supplementary material available at 10.1007/s12035-025-04719-6.

## Introduction

Epilepsy, one of the most persistent diseases of the brain, affects more than 70 million individuals globally and is characterized by recurrent and unpredictable seizures [1, 2]. Although the cause of epilepsy in many patients is unknown, any factor that can affect the function of the brain, such as stroke, traumatic brain injury, infection, genetic mutations, and autoimmune diseases, can cause epilepsy [3]. Temporal lobe epilepsy (TLE) is a common type of epilepsy in which seizures originate in the temporal lobe of the brain [4]. TLE generally arises because of an injury, such as a brain insult [5]. Epileptogenesis, a response to triggering factors, describes the process by which a previously normal brain network becomes functionally altered, resulting in increased seizure susceptibility and a higher likelihood of spontaneous recurrent seizures (SRSs) [6, 7]. Epileptogenesis consists of three phases: (1) the acute phase, which occurs immediately following the initial brain insult (such as a stroke or traumatic brain injury); (2) the latent phase, in which there are molecular and cellular changes triggered by the damage but no epileptic seizures; and (3) the chronic phase, in which SRSs occur and the brain has sufficient epileptogenic changes to produce seizures without an initial epileptogenic insult [8]. Currently, a biomarker that can represent each phase of epileptogenesis has not been identified. An electroencephalogram (EEG) is considered the standard diagnostic tool for epilepsy, but it cannot accurately predict the likelihood of epilepsy after a brain injury [9, 10]. Regarding TLE treatment, the primary approach is the usage of anti-seizure medications that act by simply suppressing seizure activity without addressing the underlying neuropathology [3, 11]. These medications have a variety of severe side effects, and unfortunately, most cases of TLE resistant to these drugs [12, 13]. Therefore, there is an urgent need to identify biomarkers associated with the phases of TLE to follow the prognosis of the disease and develop new anti-epileptogenic strategies.

The metabolome is defined as the array of small molecules found within an organism or within specific samples of that organism, such as body fluids, cell extracts, or tissues. The metabolome acts as a chemical signature of a biological system, reflecting its present state and offering key information about its physiological and pathological states. Metabolomics is an evolving approach that involves the investigation of comprehensive changes in the various metabolites within a sample and subsequent extensive data analysis and bioinformatics [14, 15]. Multiple lines of evidence indicate that the etiology of several neurological disorders is based on metabolic impairment [16, 17]. It has been reported that epilepsy can also be caused by a disruption in metabolism [18, 19]. It has also been noted that epileptic seizures can lead to alterations in metabolic processes [19]. Based on the information available in the literature, it is believed that metabolomics could serve as a swift diagnostic aid for epilepsy and potentially play a role in identifying targets for future anti-seizure therapies [14].

Nuclear magnetic resonance (NMR) spectroscopy provides the ability to gather data on numerous metabolites in biological fluids through a single experiment. Unlike other analytical methods, NMR spectroscopy possesses distinct qualities that render it applicable for analyzing mixtures of metabolites. It enables the accurate detection and quantification of a broad spectrum of metabolites that contain hydrogen, even in complex biological fluids at micromolar levels. Additionally, NMR spectroscopy is recognized as a non-invasive technique that requires minimal handling and preprocessing time [20]. The use of NMR spectroscopy to analyze plasma samples is crucial for medical diagnostics and biomarker discovery. The accessibility and non-invasiveness of these biological fluids make them an ideal choice for frequent and widespread testing. Thus, NMR’s strong metabolic profiling capacity for biological samples can improve our knowledge on the mechanisms of disease and provide a roadmap for personalized medicine.

The present study used ^1^H-NMR to analyze the metabolomics profiles of plasma samples from rats with TLE representing the defined phases of epileptogenesis. The primary goal of this research was to identify feasible biomarkers that can distinguish and reflect different time points of epileptogenesis. The detection of these indicators is crucial for identifying markers that can predict disease onset, progression, and severity, as well as suggesting metabolic pathways that could be utilized for intervention.

## Materials and Methods

**Fig. 1:**
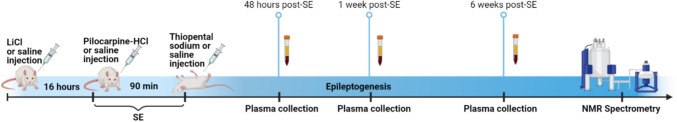
A targeted metabolomics approach using NMR spectrometry was employed to study the plasma metabolic profile during epileptogenesis in a lithium–pilocarpine induced rat model of TLE at three different time points: 48 h post-SE (SE-48 h, *n* = 5; C-48 h, *n* = 3), 1 week post-SE (SE-1wk, *n* = 5; C-1wk, *n* = 7), and 6 weeks post-SE (SE-6wk, *n* = 8; C-6wk, *n* = 6). The scheme was created using BioRender (https://biorender.com)

This study aimed to identify metabolic alterations during epileptogenesis by examining the plasma samples of rats after exposure to an initial epileptogenic injury. For this purpose, plasma samples were measured by ^1^H-NMR spectrometry for an associated screening panel including 41 metabolites. A scheme for experimental procedures is presented in Fig. 1.

### Animals

Young male Sprague–Dawley weighing 250–450 g were obtained from Acibadem University Experimental Animals Research Center (Istanbul, Türkiye). Rats were habituated to the animal facility 7 days prior to the commencement of the experimental protocol. All rats were housed at room temperature (24 ± 1°C) and maintained under a 12-h/12-h light/dark cycle (lights on at 07.00 am) with food and water available ad libitum. All protocols were approved by the Local Ethics Committee on Experimental Animal Research of Acibadem University (Approval number: HDK-2022/80). All treatments were in accordance with the ARRIVE Guidelines and the Guide for the Care and Use of Laboratory Animals Eighth Edition [21]. All precautions were taken to avoid animal suffering at each stage of the experiment.

### Chemicals

Lithium chloride, methylscopolamine, and pilocarpine hydrochloride were acquired from Sigma–Aldrich^®^ (St Louis, MO, USA). Additionally, thiopental sodium was obtained from Ibrahim Etem, part of the Menarini Group in Türkiye. For administration, 3 mEq/kg lithium chloride was mixed with injectable water and administered intraperitoneally (IP). Both 1 mg/kg methyl scopolamine and 20 mg/kg pilocarpine hydrochloride were prepared in a solution of 0.9% saline and administered IP. Similarly, 30 mg/kg thiopental sodium was prepared in injectable water and administered IP. The drug doses administered were selected according to prior studies [22, 23].

### Induction of Epileptogenesis, Group Size, and Sample Collection

In this study, we established a TLE model in rats by inducing SE through low-dose recurrent intraperitoneal injections of lithium chloride–pilocarpine hydrochloride [23]. All rats in the experimental groups received pilocarpine hydrochloride (20 mg/kg; IP) 20 h after 3 mEq/kg lithium chloride IP injection. The injection of 20 mg/kg pilocarpine was repeated every 30 min until SE occurred for a maximum of 5 doses. Methylscopolamine (1 mg/kg; IP) was injected 30 min before the first dose of pilocarpine hydrochloride to prevent peripheral cholinergic effects. Animals were behaviorally evaluated for SE according to the Racine scale [24]. To reduce mortality, 30 mg/kg thiopental sodium was injected IP 90 min after the onset of SE for a maximum of two doses separated by 10 min if necessary.

To identify metabolic alterations in the plasma of rats after the induction of epileptogenesis, we analyzed three different time points of epileptogenesis. The time points reflecting the different phases of epileptogenesis were selected by considering previous research [9, 25, 26]. For this purpose, blood samples were collected at 48 hours, 1 week and 6 weeks respectively, after SE. For the metabolomics experiments, the groups were composed of 3–8 animals as follows: 48-h controls (C-48h; *n* = 3), 48-h post-SE (SE-48h; *n* = 5), 1-week controls (C-1wk; *n* = 7), 1-week post-SE (SE-1wk; *n* = 5), 6-week controls (C-6wk; *n* = 6), and 6-week post-SE (SE-6wk; *n* = 8).

To obtain blood samples reflecting different phases of epileptogenesis, the venous blood of rats was collected by the cardiac puncture method under anesthesia with heparin-washed syringes and transferred into sterile, EDTA-filled blood collection tubes. The collected blood was immediately processed and centrifuged twice at 2500 × *g* for 15 min at 4°C to obtain plasma. Similarly, the isolated plasma samples were flash-frozen in liquid nitrogen and stored at −80°C for later analysis [27].

### NMR Sample Preparation and Experiments

Prior to NMR analysis, plasma samples stored at − 80 °C were thawed at 4 °C. Following a brief vortex, the samples were centrifuged at 14,000 × *g* for 5 min at 4 °C, after which the supernatant was separated. The obtained supernatant was then mixed with an equal volume (300 µL) of buffer solution (directly sourced from Bruker) in an Eppendorf tube. This mixture (600 µL) was subsequently transferred into 5-mm SampleJet NMR tubes for further analysis [28, 29]. Spectroscopic analyses were conducted using a Bruker Avance III HD series spectrometer operating at a frequency of 600 MHz. The spectrometer was equipped with a 5-mm broadband inverse probe and further enhanced by the integration of the Bruker SampleJet robotic system (regulated at a temperature of 5 °C) for sample cooling. We initiated the analyses after following a rigorous calibration procedure according to a protocol described in the literature [30]. To obtain the reference standards, we applied the “Electronic REference To access In vivo Concentrations (ERETIC) method” [29]. ^1^H NOESY spectra were utilized within the provided B.I.QUANT-PS™ (B.I.: Bruker BioSpin GmbH, Ettlingen, Germany) method to generate a prepared dataset for automated metabolite annotation and quantification for 41 disease-associated small metabolites [28, 29, 31].

### Statistical Analysis

Statistical analysis of the metabolites was performed using MetaboAnalyst 6.0 (www.metaboanalyst.ca, accessed multiple times in January–February 2024) [32]. Prior to analyzing the ^1^H-NMR–based metabolomics data, we conducted data filtering and data integrity checks to verify the completeness and accuracy of the essential data, which included two categories, ensure non-negative values for compound concentrations or peak intensities, and address any gaps in the data. Data normalization was performed using the normalization module in MetaboAnalyst 6.0. The data were subjected to logarithmic transformation using base 10 and automatically scaled to achieve normalization. Multivariate analysis was conducted using the orthogonal partial least squares discriminant analysis (OPLS-DA) model to explore the variations between the TLE model groups and their associated control groups. Furthermore, univariate analysis was conducted as part of the exploratory data analysis process. *p* ≤ 0.05 was considered statistically significant at this point. An analysis of fold changes (FCs) with a cutoff of 1.5 was performed to pinpoint potential metabolites associated with epileptogenesis. The cutoffs were chosen according to previous research [9]. To identify key features based on their biological and statistical relevance, volcano plot analysis was utilized applying an FC criterion (x) of 1.5 and a *t*-test threshold (y) of 0.05. Therefore, variables were considered significantly different if FC ≥ 1.5 between groups and *p* ≤ 0.05, except where specified differently in the study. For the metabolic pathway analysis, MetaboAnalyst 6.0 and the KEGG metabolic pathway database (*Rattus norvegicus*) were used.

## Results

### The Overall Metabolic Changes in Plasma

**Fig. 2:**
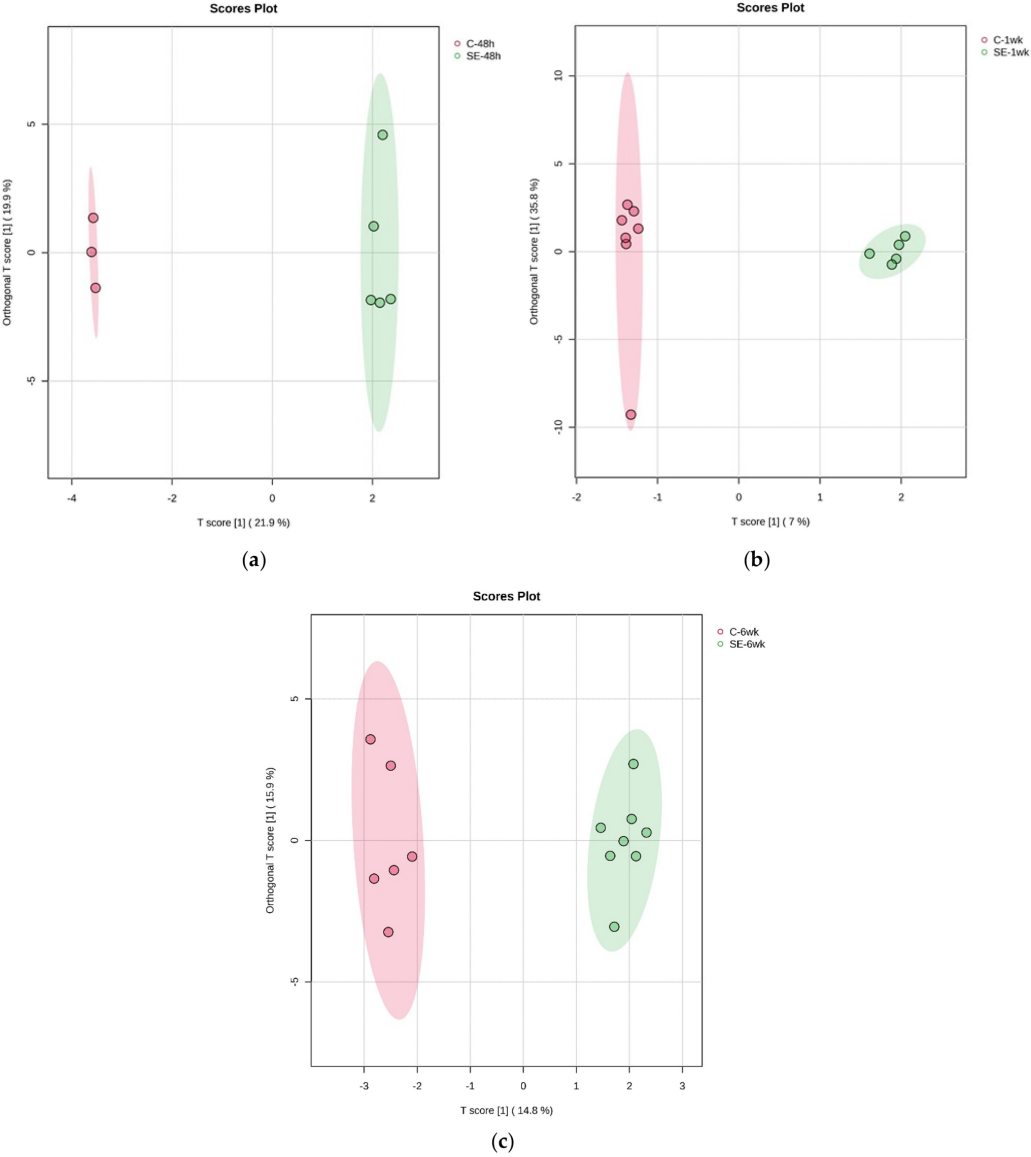
The score plot of the two-component OPLS-DA model for three different time points of epileptogenesis in plasma samples. (**a**) NMR data for C-48 h vs. SE-48 h, (**b**) C-1wk vs. SE-1wk, and (**c**) C-6wk vs. SE-6wk

The OPLS-DA model displayed significant differences in three different time points of epileptogenesis, namely between the SE-48h and C-48h groups (orthogonal T score = 19.9%, T score = 21.9%, Fig. 2a), SE-1wk and C-1wk groups (orthogonal T score = 35.8%, T score = 7%, Fig. 2b) and SE-6wk and C-6wk groups (orthogonal T score = 15.9%, T score = 14.8%, Fig. 2c).

### The Alteration of Specific Metabolites in Plasma

Metabolites play crucial roles in comprehending the complex biochemical processes involved in health and disease. To explore potential changes in the plasma metabolic profile during epileptogenesis, we compared the SE and control groups. The dataset was subjected to univariate analysis for this purpose. Student’s *t*-test and FC analyses (*p* ≤ 0.05 and FC ≥ 1.5) were used to verify the significantly different metabolic features between the SE and control groups.

**Fig. 3:**
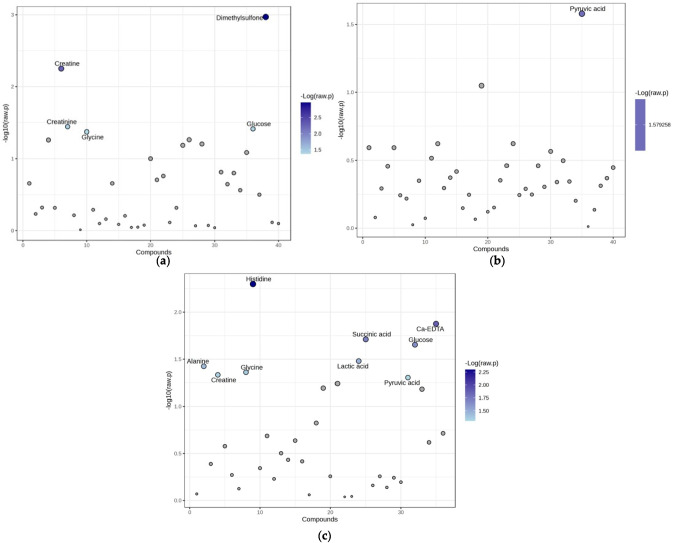
The significantly altered metabolites in plasma samples. The *t*-test statistics for NMR data for (**a**) C-48 h vs. SE-48 h, (**b**) C-1wk vs. SE-1wk. and (**c**) C-6wk vs. SE-6wk

According to the *t*-test results, statistically significant decreases were observed in dimethyl sulfone (DMSO_2_), creatinine, and glucose concentrations in the SE-48h group compared to the C-48h group, whereas creatine and glycine concentrations were increased (Fig. 3a). In the SE-1wk group compared to the C-1wk group, the only finding was a significant decrease in the pyruvic acid concentration (Fig. 3b). In the SE-6wk group, histidine, Ca^+2^-EDTA, and glucose concentrations were significantly decreased, whereas succinic acid, lactic acid, alanine, glycine, creatine, and pyruvic acid concentrations were increased (Fig. 3c).

**Fig. 4:**
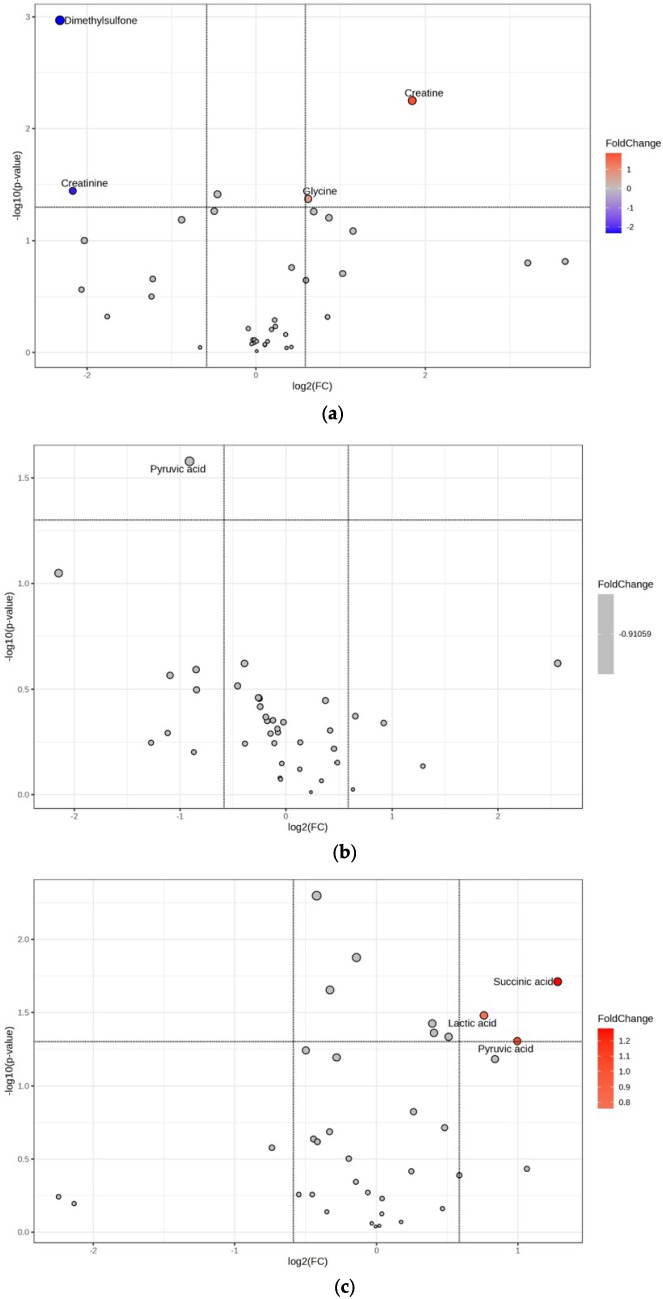
The volcano plot analysis of NMR plasma metabolites data for (**a**) C-48 h vs. SE-48 h, (**b**) C-1wk vs. SE-1wk, and (**c**) C-6wk vs. SE-6wk

FC analysis with a cutoff of 1.5 was performed to identify the metabolites potentially associated with epileptogenesis. The metabolites exhibiting FC ≥ 1.5 are listed in Tables S1, S2, and S3 for the different time points reflecting 48h, 1 week and 6 weeks post-SE, respectively. Furthermore, volcano plot analysis was performed to check the significance of these potential metabolites associated with different periods of epileptogenesis. Figure 4a illustrates that in the SE-48 h group compared to its control group, DMSO_2_ and creatinine concentrations were decreased, whereas creatine and glycine concentrations were increased. Concerning the 1-week post-SE term of epileptogenesis, the SE-1wk group exhibited a slight increase in the pyruvic acid concentration compared to its control group (Fig. 4b). The SE-6wk group featured significant increases in the concentrations of the succinic acid, lactic acid, and pyruvic acid based on volcano plot analysis (Fig. 4c). Table 1 lists the metabolites that were significantly associated with epileptogenesis according to the analyses and their corresponding statistical results. Changes in the metabolic profile mainly occurred at 48 h and 6 weeks post-SE. At 6 weeks post-SE in particular, all metabolic features were significantly increased (Fig. 4a and Table 1).

**Table 1:** The significantly changed plasma metabolites in TLE-induced rats during three different time points of epileptogenesis

Groups	Metabolites	FC	log2(FC)	p	− log10(P)	Increased/Decreased
SE-48 h vs. C-48 h	DMSO_2_	0.2	− 2.321	0.001	2.969	Decreased
	Creatinine	0.222	− 2.169	0.036	1.442	Decreased
	Glycine	1.535	0.618	0.042	1.372	Increased
	Creatine	3.594	1.845	0.005	2.250	Increased
SE-1wk vs. C-1wk	Pyruvic acid	1.879	0.910	0.026	1.579	Increased
SE-6wk vs. C-6wk	Lactic acid	1.692	0.759	0.033	1.479	Increased
	Pyruvic acid	1.992	0.994	0.049	1.304	Increased
	Succinic acid	2.429	1.280	0.019	1.710	Increased

### The Metabolic Pathway Analysis of the Plasma Metabolites

Metabolic pathway analysis was conducted using MetaboAnalyst 6.0 and the KEGG metabolic pathway database. Table 2 lists metabolites that displayed significant changes (meeting the criteria of *p* ≤ 0.05 and FC ≥ 1.5), as well as the metabolic pathways significant at the p ≤ 0.05 level. Significant associations between metabolites and pathways were observed in both terms of 48h and 6 weeks post-SE. However, no significant metabolite-based pathway effects were found 1 week after SE.

**Table 2:** The significantly changed plasma metabolites and metabolic pathways in TLE-induced rats during three different time points of epileptogenesis

Pathway Name (KEGG) (*Rattus norvegicus*)	SE-48 h vs. C-48 h		SE-1wk vs. C-1wk		SE-6wk vs. C-6wk	
	*p*	Metabolites	*p*	Metabolites	p	Metabolites
Glycine, serine, and threonine metabolism	0.015	Glycine Creatine				
Primary bile acid biosynthesis	0.042	Glycine				
Pyruvate metabolism					0.021	Lactic acid Pyruvic acid
Glycolysis/gluconeogenesis					0.021	Lactic acid Pyruvic acid
Arginine and proline metabolism					0.029	Pyruvic acid

#### Glycine, Serine, and Threonine Metabolism

There were significant increases in the concentrations of glycine and creatine metabolites 48h post-SE. Pathway analysis, conducted independently of the individual metabolite analysis, revealed that the “Glycine, serine, and threonine metabolism” pathway was statistically significant. It is noteworthy that glycine and creatine metabolites are involved in this pathway. No significant changes in metabolites were found for the glycine, serine, and threonine metabolism pathway during the latent and chronic phases.

#### Primary Bile Acid Biosynthesis

Pathway analysis conducted independently of the individual metabolite analysis revealed significant changes in the “primary bile acid biosynthesis” pathway between the SE-48h and C-48h groups. It is remarkable that only glycine was found to be involved in this pathway among the metabolites that were significant according to our criteria. The primary bile acid biosynthesis pathway did not exhibit any significant metabolite alterations at 1 week and 6 weeks post-SE.

#### Pyruvate Metabolism

The analysis revealed notable alterations in pyruvate metabolism, specifically in the levels of lactic acid and pyruvic acid metabolites, 6 weeks following SE. Conversely, no significant metabolite changes related to this pathway were observed in the terms of 48 h and 1 week post-SE.

#### Glycolysis/Gluconeogenesis

Significant alterations were identified in the glycolysis/gluconeogenesis metabolic pathway, which were associated with elevated levels of lactic acid and pyruvic acid metabolites at 6 weeks post-SE. Nevertheless, no substantial alterations were observed in the glycolysis/gluconeogenesis pathways over the course of 48 h and 1 week of epileptogenesis.

#### Arginine and Proline Metabolism

From the metabolites that were identified as significantly altered according to the pre-established criteria (*p* ≤ 0.05 and FC ≥ 1.5), only pyruvic acid demonstrated an association with arginine and proline metabolism at the 6 weeks post-SE. The level of pyruvic acid increased in 6 weeks post-SE rats compared to that in the healthy controls.

## Discussion

The process of epileptogenesis, which is responsible for the development of epilepsy, begins prior to the occurrence of the first seizure. Identifying biomarkers is key to understanding the transition from a pre-epileptic to an epileptic state and could aid in developing therapies that target the root causes of TLE, rather than just managing symptoms. Current research lacks molecular markers to assess epileptogenic risk, emphasizing the need for blood-based biomarkers for early detection.

The dynamic nature of the latent period in patients with TLE, which varies significantly among individuals, necessitates the use of reliable animal models for in-depth research. Prior studies, including those by Heischmann et al. and Meier et al., explored metabolic dysregulation in epilepsy models using different analytical techniques [9, 33]. Currently, no study in the literature has ^1^H-NMR spectroscopy, which has been proven to be an effective method for analyzing molecular structures without sample degradation, to conduct a comprehensive analysis of a broad range of metabolites and their associated metabolic pathways in plasma samples across different time points of epileptogenesis using the lithium-pilocarpine-induced TLE rat model, which closely mimics TLE. This research represents a novel approach to investigating metabolites in detail, with the aim of identifying potential biomarkers for each phase of epileptogenesis in the lithium-pilocarpine-induced SE model. The results demonstrated notable alterations in plasma metabolites following SE, with the most pronounced changes occurring during the 48 h and 6 weeks post-SE periods. In comparison, the rats in the 1 week post-SE group exhibited comparatively fewer alterations.

In our study, we identified DMSO_2_ as a key metabolite with significantly lower plasma concentrations in the acute epileptogenesis phase (48 h post-SE) compared to controls. DMSO_2_, a popular dietary supplement in alternative medicine, is the primary metabolite of dimethyl sulfoxide (DMSO) produced via methanethiol metabolism by intestinal bacteria and endogenous human enzymes [34, 35]. While not directly linked to epilepsy, it has shown anti-inflammatory and antioxidant effects in other disease models like Alzheimer's [36]. DMSO was demonstrated to have both seizure-inducing and anti-seizure effects depending on the dose in various seizure and epilepsy models [37, 38]. Moreover, the anti-inflammatory and antioxidant properties of the primary metabolite of DMSO, DMSO_2_ may confer anti-epileptogenic benefits. Its capacity to cross the blood–brain barrier enhances its potential in addressing epileptogenesis. The presence of DMSO_2_ during the acute time point could highlight its potential as a valuable biomarker for this stage, and its supplementation might support anti-epileptogenic strategies.

Epilepsy alters cellular energy metabolism, increasing ATP demand during seizures. Creatine, produced in the kidneys and liver from amino acids, aids in ATP regeneration and is transported to the brain and other tissues [39]. The findings of our study indicate that plasma creatine levels in rats are elevated during the 48-h period following SE, which may be indicative of a compensatory response to higher energy needs. Similar increases have been observed in patients with schizophrenia [40]. External creatine administration has been demonstrated to delay seizures and support GABAergic neurons in various models [41–43]. Increased plasma levels might also reflect impaired creatine transport across the blood–brain barrier (BBB) mediated by the creatine transporter protein (CRT), and 98% of the BBB lacks CRT [44]. Mutations in the CRT gene are linked to neurological impairments and seizures [45]. Thus, seizures can disrupt BBB and CRT function, leading to creatine imbalances. The exact role of creatine in the pathophysiology of epilepsy remains unclear. Further studies are needed to explore fluctuations in creatine levels during epileptogenesis and its effects on disease progression or treatment responses, potentially enhancing our understanding of epilepsy’s pathophysiology and treatment.

The creatine cycle ends with the conversion to creatinine, which is excreted in urine [44]. Creatinine also forms spontaneously from creatine via phosphocreatine conversion. In our study, plasma creatinine levels in rats during the 48-h period following SE were significantly lower than those in the controls. The low creatinine concentration despite high plasma creatine levels is probably attributable to the production of creatinine from creatine at a constant rate, the efficient renal clearance of creatinine, and the utilization of creatine by tissues. The body’s regulatory mechanisms ensure efficient removal of creatinine as a waste product while making creatine available for metabolic needs. The literature suggests creatinine as a potential biomarker for neurodegenerative diseases such as dementia and Parkinson’s disease [46–48]. In line with the previous research, a reduction in plasma creatinine levels at 48 h post-SE offers support for its potential as a biomarker for the onset of epileptogenesis. On the other hand, creatinine’s reported immunosuppressive and antibacterial effects suggest it may have roles beyond waste elimination, deserving further investigation [49, 50].

Serving as a neurotransmitter glycine, predominantly inhibits neuronal activity in various brain regions. In addition, it plays an essential role in regulating gene expression, determining protein structure and function, and influencing a broad range of biological processes [51]. In the present study, a notable increase in plasma glycine levels was observed in rats 48 h following SE. Glycine interacts with a variety of endogenous targets, including glycine receptors (GlyRs), N-methyl-d-aspartate (NMDA) receptors, G protein-coupled receptor family C group 6 (GPRC6), and glycine transporters 1 and 2 (GlyT1 and GlyT2). These interactions result in different neuromodulatory effects: GlyR binding leads to inhibition, while NMDA receptor interaction induces excitation [51]. Increased GlyT1 expression has been observed in the epileptic hippocampus of both rats and humans with TLE, and inhibiting GlyT1 raises the seizure threshold [52–54]. Moreover, GlyT1 inhibitors have also been effective in reducing chronic seizures in mouse models [55]. Recent studies indicate that glycine-mediated activation of non-classical NMDA receptors may compromise BBB integrity and contribute to epileptic activity during the acute phase [56]. Furthermore, autoantibodies against GlyRs have been found in epilepsy patients, potentially reducing glycine’s inhibitory effect, making them a possible target for future anti-epileptogenic strategies [57]. Thus, autoantibodies against GlyR receptors could represent an interesting research topic for understanding epileptogenesis and developing novel anti-epileptogenic strategies. On the other hand, the role of GPRC6 in epilepsy remains unclear. The elevated glycine levels observed in the acute phase are consistent with abnormalities in GlyR and GlyT expression, which suggests that glycine may serve as a potential biomarker for epileptogenesis. Additionally, glycine plays a pivotal role in primary bile acid synthesis, which was notably altered 48 h after SE, potentially affecting bile acid conjugation processes [58]. Investigating glycine and its targets, such as GlyT and GlyR, is critical for identifying biomarkers and developing anti-epileptogenic therapies.

Our study revealed significant metabolic differences in SE-48 h group rats, particularly in the metabolism of glycine, serine, and threonine, compared to controls. This pathway is associated with oxidative stress and inflammation and has been linked to febrile seizures in children [59]. A recent review on epilepsy and metabolomics highlighted the disruption of this pathway in epilepsy, suggesting its role in understanding the disease and identifying therapeutic targets [14]. Elevated glycine levels in the cerebrospinal fluid (CSF) of epilepsy patients further implicate this pathway [60]. Furthermore, our findings revealed that DMSO2, which exhibited considerable fluctuation at 48 h post-SE, is known to be involved in sulfur metabolism and may potentially interact with glycine, serine, and threonine metabolism. Our pathway analysis indicates that these metabolites undergo significant alteration during the acute period, specifically 48 h following SE. This finding aligns with the existing literature, suggesting that these metabolites may play a pivotal role in the onset of epileptogenesis subsequent to initial injury.

Mitochondrial dysfunction and changes in energy metabolism associated with conditions such as traumatic brain injury and seizures highlight the critical role of pyruvic and lactic acid in providing energy, particularly to cells in the nervous system. Mitochondrial dysfunction and altered energy metabolism in conditions like traumatic brain injury and seizures underscore the importance of pyruvic and lactic acid in supplying energy, especially to nervous system cells [61]. Pyruvic acid plays a pivotal role as a substrate in the tricarboxylic acid (TCA) cycle, where it is converted into acetyl-CoA, thereby facilitating ATP production [62]. Numerous studies demonstrated that pyruvic acid provides significant neuroprotective benefits in various neurological diseases and animal models, including stroke, traumatic brain injury, and hypoglycemia [63–65]. In addition, pyruvic acid can be converted to lactic acid by LDH, an enzyme linked to disease and tissue damage. Increased LDH activity has been observed in specific epileptic kindling models [66]. Aside, lactic acid, once considered a metabolic byproduct, is currently recognized for its role in energy metabolism. The results of our study indicated that pyruvic acid levels in the plasma of rats exhibited a significant elevation during the period of epileptogenesis, at both the 1 week and 6 weeks’ time points following the occurrence of SE. In contrast, lactic acid levels demonstrated a notable increase only at the six-week mark following SE. This rise in pyruvic and lactic acids suggests hypoxia, increased glycolysis, and mitochondrial dysfunction. Moreover, increased muscle activity during chronic seizures may contribute to lactic acid accumulation. Consistent with our findings, sustained increases in lactic acid levels have been observed in both epilepsy patients and models [67–70], with elevated levels reported in the hippocampus, CSF, and blood immediately after seizures [71–73]. Considering these insights, the transportation of pyruvic acid and lactic acid in and out of brain cells plays a crucial role in preserving the balance of energy metabolism. The transport of these acids is facilitated by monocarboxylate transporters (MCTs). Changes in MCT expression have been noted in epilepsy models, making them potential therapeutic targets [74]. The inhibition of lactic acid transport through MCT1/MCT2 has been shown to reduce seizures in mice, highlighting lactate’s role as a primary neuronal fuel [75]. Furthermore, a reduction in MCT4 expression has also been observed in TLE in both humans and the pilocarpine rat model [76]. Recent studies suggest that lactate’s role extends beyond metabolism, acting as a signaling molecule involved in epigenetic regulation through lactylation, which influences gene expression and inflammatory responses [77–79]. Lactylation, which is widespread among brain cells, is regulated by neural activity and stress, markedly influencing cellular functions [80]. In Alzheimer’s disease, the lactate-driven transition from oxidative phosphorylation to glycolysis in microglia triggers pro-inflammatory signaling and microglial dysfunction facilitated by a feedback mechanism involving lactate-induced lactylation [81]. Similarly, in sepsis, lactate absorption by macrophages results in the lactylation of the nuclear protein HMGB1, enhancing endothelial permeability via its pro-inflammatory properties. Although the role of lactylation in epilepsy is underexplored, the lactylation of proteins like HMGB1, a biomarker in epilepsy, suggests its relevance in epileptogenesis [82, 83]. In this context, the complex relationship among metabolism, epigenetics, and post-transcriptional regulation represents an intriguing but understudying area that could elucidate cellular mechanisms. The dynamic interplay between pyruvic and lactic acids during epileptogenesis highlights the potential of monitoring these metabolites and MCT expression to understand disease progression. MCTs have emerged as promising therapeutic targets to delay or inhibit epileptogenesis, making them crucial for future epilepsy research and treatment strategies.

Mitochondria are essential for cellular function, particularly in the management of intermediary metabolism and bioenergetics. The complexity of mitochondrial dysfunction in chronic epilepsy is a challenge in determining whether mitochondrial oxidative stress is a precursor or consequence of seizures. Succinic acid serves as a pivotal link between various metabolic pathways, playing a critical role by pooling catabolic molecules and triggering anabolic processes [84]. In particular, it is a key player in the TCA cycle and the γ-aminobutyric acid (GABA) shunt. The present study revealed a significant increase in plasma succinate levels in SE-6wk rats. Recent findings indicate that succinate accumulation may result from reverse catalysis by succinate dehydrogenase (SDH), which typically converts succinate to fumarate. During periods of ischemia, SDH catalyzes a reverse reaction, resulting in the generation of ROS upon reperfusion and subsequent neuronal damage [85]. Consistent with our results, another study found elevated succinate levels in the hippocampus of rats with kainic acid-induced SE, primarily due to reverse SDH activity [86]. Similarly, increased succinic acid levels were detected in the CSF of dogs with idiopathic epilepsy [87]. Succinic acid also acts as a partial NMDA agonist, enhancing post-synaptic excitability and triggering convulsions [88]. In addition, succinate is closely linked to GABA metabolism, as GABA is converted into succinic semialdehyde, then oxidized to succinate by succinic semialdehyde dehydrogenase (SSADH). In the literature, a study involving the *ALDH5A1* gene, which encodes this enzyme, suggested that high-stress conditions like SE could lead to significant activation in the *ALDH5A1* promoter region, resulting in increased production of the ALDH5A1 protein [89]. It is thus possible that under conditions of elevated stress, such as those observed in SE, the elevated levels of ALDH5A1 protein may lead to enhanced synthesis of succinic acid, resulting in its accumulation in plasma. Furthermore, a study conducted on rat brain mitochondria indicated that damage-causing lipid peroxidation resulted in the inhibition of the TCA cycle enzyme SSADH by lipid peroxidation products [90]. This resulted in a reduction in the flow of the TCA cycle and a decrease in GABA clearance. Additionally, electrophysiological data indicates that this could lead to the excitatory transformation of extracellular GABA due to the functional impairment of ligand-gated chloride channels (GABA_A_), which may contribute to further seizure-related damage [91]. Besides, succinic acid is also involved in protein succinylation, a post-translational modification, and acts as a chemical messenger, though its regulatory effects remain unclear [84]. These findings suggest that succinate may have different roles in physiological versus pathological processes and could disrupt the GABA-glutamate balance, exacerbating SE pathology. Given its elevated levels in various epilepsy models and samples, succinic acid shows potential as a biomarker for monitoring epileptogenesis.

The significant metabolic changes observed during the latent and chronic phases of epileptogenesis were particularly evident in the TCA cycle. Consistent with our findings, impaired TCA cycle function has been noted in the cerebral cortex and hippocampus in mice with SRSs, suggesting that targeting mitochondrial dysfunction may offer a promising approach for drug-resistant epilepsy [92]. Our analysis of the TCA cycle revealed a key interaction with glycine, serine, and threonine metabolism. Although the precise mechanisms underpinning this interaction remain somewhat elusive, it is apparent that anaplerotic substrates such as serine, threonine, and glycine, which are converted into pyruvate, play a crucial role in supplementing TCA cycle metabolites [93]. This process is crucial for maintaining TCA cycle homeostasis and reflects the metabolic flexibility required to sustain cellular function and energy production. The relationship between the glycine, serine, and threonine pathway, which appears to be significant in the acute phase, and the TCA cycle, important in the chronic phase, warrants further investigation. Exploring this interaction could offer new insights into metabolic regulation and potential therapeutic targets for epileptogenesis.

The current findings highlight the significant impact of alterations in pyruvic acid and lactic acid levels on glycolysis/gluconeogenesis and pyruvate metabolism pathways, as observed 6 weeks following the occurrence of SE. Pyruvate and lactate are linked to each other both within the Cori cycle, as needed in gluconeogenesis pathway, and in anaerobic metabolism with the conversion of pyruvate to lactate in the absence of sufficient oxygen. In particular, lactate, as an alternative energy substrate, can be accumulated by brain tissues under stressful conditions such as epileptogenesis [94]. The results of our study demonstrated that alterations in pyruvate and lactate metabolite levels were only statistically significant in the glycolysis/gluconeogenesis and pyruvate metabolism pathways during the 6-week period following SE. These findings highlight the importance of these metabolites in selecting the appropriate treatment or follow-up period. In addition, pyruvic acid can participate in arginine and proline metabolism. In this metabolic pathway, pyruvic acid participates in arginine synthesis, which is important for detoxification of ammonia, indirectly through the TCA cycle. It can also contribute to proline synthesis indirectly through the pentose phosphate pathway, producing NADPH metabolites that are subsequently used for detoxification and cellular redox balance. In neurological disorders such as epilepsy, oxidative stress and neuroinflammation occur, and arginine/proline metabolite production is increased to combat this metabolic dysregulation and excessive neuronal hyperexcitability [95].

In light of the aforementioned points, it is notable that pyruvic acid is present both in the period 1 week after SE and in the subsequent period of 6 weeks after SE. Pyruvic acid’s role in epileptogenesis is closely linked to its function in energy metabolism, its antioxidant properties and its potential for neuroprotection. Dysregulation of pyruvate metabolism can result in metabolic distress, increased oxidative stress, and neuronal damage, all of which contribute to the development and progression of epilepsy. Further research is required to gain a deeper understanding of the precise mechanism by which pyruvic acid is involved in epileptogenesis and to determine how modulation of pyruvate metabolism could be employed as a therapeutic intervention in epilepsy.

## Conclusion

The mechanisms and biomarkers associated with epileptogenesis have not been extensively explored, but our findings demonstrate the potential utility of further research in this area. Our investigation successfully uncovered viable biomarkers by examining the plasma metabolomic profile of rats with induced TLE across three distinct time points during epileptogenesis. Comprehensive pathway analysis and examination of significant metabolites provided insight into potential metabolic pathways involved in the development of epilepsy. The usefulness of plasma metabolomics in identifying biomarkers for epileptogenesis was highlighted, paving the way for a better understanding of the mechanisms driving SE and opening avenues for the development of targeted therapeutic interventions. Further research is necessary to evaluate the roles of these metabolites and their associated molecular pathways in epileptogenesis within human cohorts before translating these findings into clinical practice.

## Supplementary Information

Below is the link to the electronic supplementary material.

Supplementary file1 (DOCX 16 KB)

Supplementary file2 (DOCX 15 KB)

Supplementary file3 (DOCX 15 KB)

## Data Availability

Enquiries about data availability should be directed to the authors.

## References

[1] Fisher RS, Acevedo C, Arzimanoglou A, Bogacz A, Cross JH, Elger CE, Engel J Jr, Forsgren L et al (2014) ILAE official report: a practical clinical definition of epilepsy. Epilepsia 55(4):475–482. 10.1111/epi.12550 24730690 10.1111/epi.12550

[2] Thijs RD, Surges R, O’Brien TJ, Sander JW (2019) Epilepsy in adults. Lancet 393(10172):689–701. 10.1016/S0140-6736(18)32596-0 30686584 10.1016/S0140-6736(18)32596-0

[3] Devinsky O, Vezzani A, O’Brien TJ, Jette N, Scheffer IE, de Curtis M, Perucca P (2018) Epilepsy. Nat Rev Dis Primers 4:18024. 10.1038/nrdp.2018.24 29722352 10.1038/nrdp.2018.24

[4] Marissal T (2021) An inventory of basic research in temporal lobe epilepsy. Rev Neurol (Paris) 177(9):1069–1081. 10.1016/j.neurol.2021.02.390 34176659 10.1016/j.neurol.2021.02.390

[5] Rowley S, Liang LP, Fulton R, Shimizu T, Day B, Patel M (2015) Mitochondrial respiration deficits driven by reactive oxygen species in experimental temporal lobe epilepsy. Neurobiol Dis 75:151–158. 10.1016/j.nbd.2014.12.025 25600213 10.1016/j.nbd.2014.12.025 PMC4465449

[6] Dudek FE, Staley KJ (2012) The time course and circuit mechanisms of acquired epileptogenesis. In: Noebels JL, Avoli M, Rogawski MA, Olsen RW, Delgado-Escueta AV (eds) Jasper's Basic Mechanisms of the Epilepsies 4th edn. Bethesda (MD), National Center for Biotechnology Information (US) 22787656

[7] Goldberg EM, Coulter DA (2013) Mechanisms of epileptogenesis: a convergence on neural circuit dysfunction. Nat Rev Neurosci 14(5):337–349. 10.1038/nrn3482 23595016 10.1038/nrn3482 PMC3982383

[8] Reddy DS (2013) Role of hormones and neurosteroids in epileptogenesis. Front Cell Neurosci 7:115. 10.3389/fncel.2013.00115 23914154 10.3389/fncel.2013.00115 PMC3728472

[9] Heischmann S, Quinn K, Cruickshank-Quinn C, Liang LP, Reisdorph R, Reisdorph N, Patel M (2016) Exploratory metabolomics profiling in the kainic acid rat model reveals depletion of 25-hydroxyvitamin D3 during epileptogenesis. Sci Rep 6:31424. 10.1038/srep31424 27526857 10.1038/srep31424 PMC4985632

[10] Drenthen GS, Jansen JFA, Gommer E, Gupta L, Hofman PAM, van Kranen-Mastenbroek VH, Hilkman DM, Vlooswijk MCG et al (2021) Predictive value of functional MRI and EEG in epilepsy diagnosis after a first seizure. Epilepsy Behav 115:107651. 10.1016/j.yebeh.2020.107651 33309424 10.1016/j.yebeh.2020.107651

[11] Chen Z, Brodie MJ, Liew D, Kwan P (2018) Treatment outcomes in patients with newly diagnosed epilepsy treated with established and new antiepileptic drugs: a 30-year longitudinal cohort study. JAMA Neurol 75(3):279–286. 10.1001/jamaneurol.2017.3949 29279892 10.1001/jamaneurol.2017.3949 PMC5885858

[12] Khoshkhoo S, Wang Y, Chahine Y, Erson-Omay EZ, Robert SM, Kiziltug E, Damisah EC, Nelson-Williams C et al (2023) Contribution of somatic Ras/Raf/mitogen-activated protein kinase variants in the hippocampus in drug-resistant mesial temporal lobe epilepsy. JAMA Neurol 80(6):578–587. 10.1001/jamaneurol.2023.0473 37126322 10.1001/jamaneurol.2023.0473 PMC10152377

[13] Lamberink HJ, Otte WM, Blümcke I, Braun KPJ (2020) Seizure outcome and use of antiepileptic drugs after epilepsy surgery according to histopathological diagnosis: a retrospective multicentre cohort study. Lancet Neurol 19(9):748–757. 10.1016/s1474-4422(20)30220-9 32822635 10.1016/S1474-4422(20)30220-9

[14] Lai W, Du D, Chen L (2022) Metabolomics provides novel insights into epilepsy diagnosis and treatment: a review. Neurochem Res 47(4):844–859. 10.1007/s11064-021-03510-y 35067830 10.1007/s11064-021-03510-y

[15] Shao Y, Le W (2019) Recent advances and perspectives of metabolomics-based investigations in Parkinson’s disease. Mol Neurodegener 14(1):3. 10.1186/s13024-018-0304-2 30634989 10.1186/s13024-018-0304-2 PMC6330496

[16] Beal MF (2004) Mitochondrial dysfunction and oxidative damage in Alzheimer’s and Parkinson’s diseases and coenzyme Q10 as a potential treatment. J Bioenerg Biomembr 36(4):381–386. 10.1023/b:Jobb.0000041772.74810.92 15377876 10.1023/B:JOBB.0000041772.74810.92

[17] Rahman S (2012) Mitochondrial disease and epilepsy. Dev Med Child Neurol 54(5):397–406. 10.1111/j.1469-8749.2011.04214.x 22283595 10.1111/j.1469-8749.2011.04214.x

[18] Scheffer IE, Berkovic S, Capovilla G, Connolly MB, French J, Guilhoto L, Hirsch E, Jain S et al (2017) ILAE classification of the epilepsies: position paper of the ILAE commission for classification and terminology. Epilepsia 58(4):512–521. 10.1111/epi.13709 28276062 10.1111/epi.13709 PMC5386840

[19] Wang D, Wang X, Kong J, Wu J, Lai M (2016) GC-MS-based metabolomics discovers a shared serum metabolic characteristic among three types of epileptic seizures. Epilepsy Res 126:83–89. 10.1016/j.eplepsyres.2016.07.003 27450370 10.1016/j.eplepsyres.2016.07.003

[20] Toczylowska B, Zieminska E, Polowy R, Olszynski KH, Lazarewicz JW (2022) NMR-based metabolomics of rat hippocampus, serum, and urine in two models of autism. Mol Neurobiol 59(9):5452–5475. 10.1007/s12035-022-02912-5 35715683 10.1007/s12035-022-02912-5

[21] Percie du Sert N, Hurst V, Ahluwalia A, Alam S, Avey MT, Baker M, Browne WJ, Clark A et al (2020) The ARRIVE guidelines 20: updated guidelines for reporting animal research. PLoS Biol 18(7):e3000410. 10.1371/journal.pbio.3000410 32663219 10.1371/journal.pbio.3000410 PMC7360023

[22] Cunha AO, Mortari MR, Carolino RO, Coutinho-Netto J, Dos Santos WF (2007) Glutamate binding is altered in hippocampus and cortex of Wistar rats after pilocarpine-induced status epilepticus. Neurosci Lett 424(1):51–54. 10.1016/j.neulet.2007.07.010 17709190 10.1016/j.neulet.2007.07.010

[23] Glien M, Brandt C, Potschka H, Voigt H, Ebert U, Löscher W (2001) Repeated low-dose treatment of rats with pilocarpine: low mortality but high proportion of rats developing epilepsy. Epilepsy Res 46(2):111–119. 10.1016/s0920-1211(01)00272-8 11463512 10.1016/s0920-1211(01)00272-8

[24] Racine RJ (1972) Modification of seizure activity by electrical stimulation. II. Motor seizure. Electroencephalogr Clin Neurophysiol 32(3):281–294. 10.1016/0013-4694(72)90177-0 4110397 10.1016/0013-4694(72)90177-0

[25] Cavalheiro EA (1995) The pilocarpine model of epilepsy. Ital J Neurol Sci 16(1–2):33–37. 10.1007/bf02229072 7642349 10.1007/BF02229072

[26] Gitaí DLG, Dos Santos YDR, Upadhya R, Kodali M, Madhu LN, Shetty AK (2020) Extracellular vesicles in the forebrain display reduced miR-346 and miR-331-3p in a rat model of chronic temporal lobe epilepsy. Mol Neurobiol 57(3):1674–1687. 10.1007/s12035-019-01797-1 31813125 10.1007/s12035-019-01797-1 PMC8011947

[27] Hanifa MA, Skott M, Maltesen RG, Rasmussen BS, Nielsen S, Frøkiær J, Ring T, Wimmer R (2020) Tissue, urine and serum NMR metabolomics dataset from a 5/6 nephrectomy rat model of chronic kidney disease. Data Brief 33:106567. 10.1016/j.dib.2020.106567 33304964 10.1016/j.dib.2020.106567 PMC7708935

[28] Beckonert O, Keun HC, Ebbels TM, Bundy J, Holmes E, Lindon JC, Nicholson JK (2007) Metabolic profiling, metabolomic and metabonomic procedures for NMR spectroscopy of urine, plasma, serum and tissue extracts. Nat Protoc 2(11):2692–2703. 10.1038/nprot.2007.376 18007604 10.1038/nprot.2007.376

[29] Crook AA, Powers R (2020) Quantitative NMR-based biomedical metabolomics: current status and applications. Molecules 25(21):5128. 10.3390/molecules25215128 33158172 10.3390/molecules25215128 PMC7662776

[30] Jiménez B, Holmes E, Heude C, Tolson RF, Harvey N, Lodge SL, Chetwynd AJ, Cannet C et al (2018) Quantitative lipoprotein subclass and low molecular weight metabolite analysis in human serum and plasma by (1)H NMR spectroscopy in a multilaboratory trial. Anal Chem 90(20):11962–11971. 10.1021/acs.analchem.8b02412 30211542 10.1021/acs.analchem.8b02412

[31] Lodge S, Nitschke P, Loo RL, Kimhofer T, Bong SH, Richards T, Begum S, Spraul M et al (2021) Low volume in vitro diagnostic proton NMR spectroscopy of human blood plasma for lipoprotein and metabolite analysis: application to SARS-CoV-2 biomarkers. J Proteome Res 20(2):1415–1423. 10.1021/acs.jproteome.0c00815 33491459 10.1021/acs.jproteome.0c00815

[32] Pang Z, Chong J, Zhou G, de Lima Morais DA, Chang L, Barrette M, Gauthier C, Jacques P et al (2021) MetaboAnalyst 5.0: narrowing the gap between raw spectra and functional insights. Nucleic Acids Res 49(W1):w388–w396. 10.1093/nar/gkab382 34019663 10.1093/nar/gkab382 PMC8265181

[33] Meier L, Bruginski E, Marafiga JR, Caus LB, Pasquetti MV, Calcagnotto ME, Campos FR (2023) Hippocampal metabolic profile during epileptogenesis in the pilocarpine model of epilepsy. Biomed Chromatogr e5820. 10.1002/bmc.5820 10.1002/bmc.5820 38154955

[34] Butawan M, Benjamin RL, Bloomer RJ (2017) Methylsulfonylmethane: applications and safety of a novel dietary supplement. Nutrients 9(3):290. 10.3390/nu9030290 28300758 10.3390/nu9030290 PMC5372953

[35] Joung YH, Darvin P, Kang DY, Sp N, Byun HJ, Lee CH, Lee HK, Yang YM (2016) Methylsulfonylmethane inhibits RANKL-induced osteoclastogenesis in BMMs by suppressing NF-kappaB and STAT3 activities. PLoS ONE 11(7):e0159891. 10.1371/journal.pone.0159891 27447722 10.1371/journal.pone.0159891 PMC4957779

[36] Abdel-Rafei MK, Thabet NM (2020) Modulatory effect of methylsulfonylmethane against BPA/γ-radiation induced neurodegenerative alterations in rats: influence of TREM-2/DAP-12/Syk pathway. Life Sci 260:118410. 10.1016/j.lfs.2020.118410 32926927 10.1016/j.lfs.2020.118410

[37] Carletti F, Ferraro G, Rizzo V, Cannizzaro C, Sardo P (2013) Antiepileptic effect of dimethyl sulfoxide in a rat model of temporal lobe epilepsy. Neurosci Lett 546:31–35. 10.1016/j.neulet.2013.04.031 23643984 10.1016/j.neulet.2013.04.031

[38] Widmann M, Lieb A, Mutti A, Schwarzer C (2023) Dimethyl sulfoxide’s impact on epileptiform activity in a mouse model of chronic temporal lobe epilepsy. Epilepsy Res 197:107235. 10.1016/j.eplepsyres.2023.107235 37797423 10.1016/j.eplepsyres.2023.107235 PMC7615238

[39] Wyss M, Kaddurah-Daouk R (2000) Creatine and creatinine metabolism. Physiol Rev 80(3):1107–1213. 10.1152/physrev.2000.80.3.1107 10893433 10.1152/physrev.2000.80.3.1107

[40] Koike S, Bundo M, Iwamoto K, Suga M, Kuwabara H, Ohashi Y, Shinoda K, Takano Y et al (2014) A snapshot of plasma metabolites in first-episode schizophrenia: a capillary electrophoresis time-of-flight mass spectrometry study. Transl Psychiatry 4(4):e379. 10.1038/tp.2014.19 24713860 10.1038/tp.2014.19 PMC4012283

[41] Kim DW, Yeo SI, Ryu HJ, Kim JE, Song HK, Kwon OS, Choi SY, Kang TC (2010) Effects of creatine and β-guanidinopropionic acid and alterations in creatine transporter and creatine kinases expression in acute seizure and chronic epilepsy models. BMC Neurosci 11:141. 10.1186/1471-2202-11-141 20979657 10.1186/1471-2202-11-141 PMC2978220

[42] Okwuofu EO, Ogundepo GE, Akhigbemen AM, Abiola AL, Ozolua RI, Igbe I, Chinazamoku O (2021) Creatine attenuates seizure severity, anxiety and depressive-like behaviors in pentylenetetrazole kindled mice. Metab Brain Dis 36(4):571–579. 10.1007/s11011-021-00684-w 33559804 10.1007/s11011-021-00684-w

[43] Andres RH, Ducray AD, Huber AW, Pérez-Bouza A, Krebs SH, Schlattner U, Seiler RW, Wallimann T et al (2005) Effects of creatine treatment on survival and differentiation of GABA-ergic neurons in cultured striatal tissue. J Neurochem 95(1):33–45. 10.1111/j.1471-4159.2005.03337.x 16045451 10.1111/j.1471-4159.2005.03337.x

[44] Ainsley Dean PJ, Arikan G, Opitz B, Sterr A (2017) Potential for use of creatine supplementation following mild traumatic brain injury. Concussion 2(2):Cnc34. 10.2217/cnc-2016-0016 30202575 10.2217/cnc-2016-0016 PMC6094347

[45] Leuzzi V, Mastrangelo M, Battini R, Cioni G (2013) Inborn errors of creatine metabolism and epilepsy. Epilepsia 54(2):217–227. 10.1111/epi.12020 23157605 10.1111/epi.12020

[46] Jiang Y, Zhu Z, Shi J, An Y, Zhang K, Wang Y, Li S, Jin L et al (2019) Metabolomics in the development and progression of dementia: a systematic review. Front Neurosci 13:343. 10.3389/fnins.2019.00343 31031585 10.3389/fnins.2019.00343 PMC6474157

[47] Tsuruoka M, Hara J, Hirayama A, Sugimoto M, Soga T, Shankle WR, Tomita M (2013) Capillary electrophoresis-mass spectrometry-based metabolome analysis of serum and saliva from neurodegenerative dementia patients. Electrophoresis 34(19):2865–2872. 10.1002/elps.201300019 23857558 10.1002/elps.201300019

[48] Yang G, Wang LZ, Zhang R, Zhang XY, Yu Y, Ma HR, He XG (2023) Study on the correlation between blood urea nitrogen, creatinine level, proteinuria and Parkinson’s disease. Neurol India 71(6):1217–1221. 10.4103/0028-3886.391388 38174461 10.4103/0028-3886.391388

[49] Leland KM, McDonald TL, Drescher KM (2011) Effect of creatine, creatinine, and creatine ethyl ester on TLR expression in macrophages. Int Immunopharmacol 11(9):1341–1347. 10.1016/j.intimp.2011.04.018 21575742 10.1016/j.intimp.2011.04.018 PMC3157573

[50] McDonald T, Drescher KM, Weber A, Tracy S (2012) Creatinine inhibits bacterial replication. J Antibiot (Tokyo) 65(3):153–156. 10.1038/ja.2011.131 22293916 10.1038/ja.2011.131

[51] Aguayo-Ceron KA, Sanchez-Munoz F, Gutierrez-Rojas RA, Acevedo-Villavicencio LN, Flores-Zarate AV, Huang F, Giacoman-Martinez A, Villafana S et al (2023) Glycine: the smallest anti-inflammatory micronutrient. Int J Mol Sci 24(14):11236. 10.3390/ijms241411236 37510995 10.3390/ijms241411236 PMC10379184

[52] Chen R, Okabe A, Sun H, Sharopov S, Hanganu-Opatz IL, Kolbaev SN, Fukuda A, Luhmann HJ et al (2014) Activation of glycine receptors modulates spontaneous epileptiform activity in the immature rat hippocampus. J Physiol 592(10):2153–2168. 10.1113/jphysiol.2014.271700 24665103 10.1113/jphysiol.2014.271700 PMC4227900

[53] Eichler SA, Kirischuk S, Jüttner R, Schaefermeier PK, Legendre P, Lehmann TN, Gloveli T, Grantyn R et al (2008) Glycinergic tonic inhibition of hippocampal neurons with depolarizing GABAergic transmission elicits histopathological signs of temporal lobe epilepsy. J Cell Mol Med 12(6b):2848–2866. 10.1111/j.1582-4934.2008.00357.x 19210758 10.1111/j.1582-4934.2008.00357.x PMC3828897

[54] Shen HY, van Vliet EA, Bright KA, Hanthorn M, Lytle NK, Gorter J, Aronica E, Boison D (2015) Glycine transporter 1 is a target for the treatment of epilepsy. Neuropharmacology 99:554–565. 10.1016/j.neuropharm.2015.08.031 26302655 10.1016/j.neuropharm.2015.08.031 PMC4655139

[55] Kalinichev M, Starr KR, Teague S, Bradford AM, Porter RA, Herdon HJ (2010) Glycine transporter 1 (GlyT1) inhibitors exhibit anticonvulsant properties in the rat maximal electroshock threshold (MEST) test. Brain Res 1331:105–113. 10.1016/j.brainres.2010.03.032 20303337 10.1016/j.brainres.2010.03.032

[56] Epping L, Schroeter CB, Nelke C, Bock S, Gola L, Ritter N, Herrmann AM, Räuber S et al (2022) Activation of non-classical NMDA receptors by glycine impairs barrier function of brain endothelial cells. Cell Mol Life Sci 79(9):ARTN479. 10.1007/s00018-022-04502-z 10.1007/s00018-022-04502-z PMC9372018 35951110

[57] Wiessler AL, Talucci I, Piro I, Seefried S, Hörlin V, Baykan BB, Tüzün E, Schaefer N et al (2024) Glycine receptor β-targeting autoantibodies contribute to the pathology of autoimmune diseases. Neurol Neuroimmunol Neuroinflamm 11(2):e200187. 10.1212/nxi.0000000000200187 38215349 10.1212/NXI.0000000000200187 PMC10786602

[58] Di Ciaula A, Garruti G, Baccetto RL, Molina-Molina E, Bonfrate L, Wang DQH, Portincasa P (2017) Bile acid physiology. Ann Hepatol 16:S4–S14. 10.5604/01.3001.0010.5493 29080336 10.5604/01.3001.0010.5493

[59] Yang L, Tian J (2023) Changes of intestinal flora in children with febrile seizure. Medicine (Baltimore) 102(20):e33730. 10.1097/md.0000000000033730 37335742 10.1097/MD.0000000000033730 PMC10194469

[60] Niu D, Sun P, Zhang F, Song F (2022) Metabonomic analysis of cerebrospinal fluid in epilepsy. Ann Transl Med 10(8):449. 10.21037/atm-22-1219 35571432 10.21037/atm-22-1219 PMC9096421

[61] Proia P, Di Liegro CM, Schiera G, Fricano A, Di Liegro I (2016) Lactate as a metabolite and a regulator in the central nervous system. Int J Mol Sci 17(9):1450. 10.3390/ijms17091450 27598136 10.3390/ijms17091450 PMC5037729

[62] Gray LR, Tompkins SC, Taylor EB (2014) Regulation of pyruvate metabolism and human disease. Cell Mol Life Sci 71(14):2577–2604. 10.1007/s00018-013-1539-2 24363178 10.1007/s00018-013-1539-2 PMC4059968

[63] Fei Y, Shi R, Song Z, Wu J (2020) Metabolic control of epilepsy: a promising therapeutic target for epilepsy. Front Neurol 11:592514. 10.3389/fneur.2020.592514 33363507 10.3389/fneur.2020.592514 PMC7753014

[64] Lee JY, Kim YH, Koh JY (2001) Protection by pyruvate against transient forebrain ischemia in rats. J Neurosci 21(20):Rc171. 10.1523/JNEUROSCI.21-20-j0002.2001 11588201 10.1523/JNEUROSCI.21-20-j0002.2001 PMC6763857

[65] Moro N, Ghavim SS, Harris NG, Hovda DA, Sutton RL (2016) Pyruvate treatment attenuates cerebral metabolic depression and neuronal loss after experimental traumatic brain injury. Brain Res 1642:270–277. 10.1016/j.brainres.2016.04.005 27059390 10.1016/j.brainres.2016.04.005 PMC4899222

[66] Suh SW, Aoyama K, Matsumori Y, Liu J, Swanson RA (2005) Pyruvate administered after severe hypoglycemia reduces neuronal death and cognitive impairment. Diabetes 54(5):1452–1458. 10.2337/diabetes.54.5.1452 15855333 10.2337/diabetes.54.5.1452

[67] Boguszewicz L, Jamroz E, Ciszek M, Emich-Widera E, Kijonka M, Banasik T, Skorupa A, Sokol M (2019) NMR-based metabolomics in pediatric drug resistant epilepsy - preliminary results. Sci Rep 9(1):15035. 10.1038/s41598-019-51337-z 31636291 10.1038/s41598-019-51337-z PMC6803684

[68] Carmody S, Brennan L (2010) Effects of pentylenetetrazole-induced seizures on metabolomic profiles of rat brain. Neurochem Int 56(2):340–344. 10.1016/j.neuint.2009.11.004 19913064 10.1016/j.neuint.2009.11.004

[69] Rabeson H, Fauvelle F, Testylier G, Foquin A, Carpentier P, Dorandeu F, van Ormondt D, Graveron-Demilly D (2008) Quantitation with QUEST of brain HRMAS-NMR signals: application to metabolic disorders in experimental epileptic seizures. Magn Reson Med 59(6):1266–1273. 10.1002/mrm.21610 18506844 10.1002/mrm.21610

[70] Wei C, Li Y, Yao H, Liu H, Zhang X, Guo R (2012) A metabonomics study of epilepsy in patients using gas chromatography coupled with mass spectrometry. Mol Biosyst 8(8):2197–2204. 10.1039/c2mb25105a 22706165 10.1039/c2mb25105a

[71] Cavus I, Kasoff WS, Cassaday MP, Jacob R, Gueorguieva R, Sherwin RS, Krystal JH, Spencer DD et al (2005) Extracellular metabolites in the cortex and hippocampus of epileptic patients. Ann Neurol 57(2):226–235. 10.1002/ana.20380 15668975 10.1002/ana.20380

[72] Chatzikonstantinou A, Ebert AD, Hennerici MG (2015) Cerebrospinal fluid findings after epileptic seizures. Epileptic Disord 17(4):453–459. 10.1684/epd.2015.0779 26575850 10.1684/epd.2015.0779

[73] Magnusson C, Herlitz J, Höglind R, Wennberg P, Edelvik Tranberg A, Axelsson C, Zelano J (2021) Prehospital lactate levels in blood as a seizure biomarker: a multi-center observational study. Epilepsia 62(2):408–415. 10.1111/epi.16806 33417237 10.1111/epi.16806 PMC7898511

[74] Leroy C, Pierre K, Simpson IA, Pellerin L, Vannucci SJ, Nehlig A (2011) Temporal changes in mRNA expression of the brain nutrient transporters in the lithium-pilocarpine model of epilepsy in the immature and adult rat. Neurobiol Dis 43(3):588–597. 10.1016/j.nbd.2011.05.007 21624469 10.1016/j.nbd.2011.05.007 PMC3726264

[75] Forero-Quintero LS, Deitmer JW, Becker HM (2017) Reduction of epileptiform activity in ketogenic mice: the role of monocarboxylate transporters. Sci Rep 7(1):4900. 10.1038/s41598-017-05054-0 28687765 10.1038/s41598-017-05054-0 PMC5501801

[76] Liu B, Niu L, Shen MZ, Gao L, Wang C, Li J, Song LJ, Tao Y et al (2014) Decreased astroglial monocarboxylate transporter 4 expression in temporal lobe epilepsy. Mol Neurobiol 50(2):327–338. 10.1007/s12035-013-8619-z 24464262 10.1007/s12035-013-8619-z

[77] Kambe Y (2022) Recent behavioral findings of pathophysiological involvement of lactate in the central nervous system. Biochim Biophys Acta Gen Subj 1866(7):130137. 10.1016/j.bbagen.2022.130137 35385782 10.1016/j.bbagen.2022.130137

[78] Muraleedharan R, Gawali MV, Tiwari D, Sukumaran A, Oatman N, Anderson J, Nardini D, Bhuiyan MAN et al (2020) AMPK-regulated astrocytic lactate shuttle plays a non-cell-autonomous role in neuronal survival. Cell Rep 32(9):108092. 10.1016/j.celrep.2020.108092 32877674 10.1016/j.celrep.2020.108092 PMC7531170

[79] Wu H, Huang H, Zhao Y (2023) Interplay between metabolic reprogramming and post-translational modifications: from glycolysis to lactylation. Front Immunol 14:1211221. 10.3389/fimmu.2023.1211221 37457701 10.3389/fimmu.2023.1211221 PMC10338923

[80] Hagihara H, Shoji H, Otabi H, Toyoda A, Katoh K, Namihira M, Miyakawa T (2021) Protein lactylation induced by neural excitation. Cell Rep 37(2):109820. 10.1016/j.celrep.2021.109820 34644564 10.1016/j.celrep.2021.109820

[81] Pan RY, He L, Zhang J, Liu X, Liao Y, Gao J, Liao Y, Yan Y et al (2022) Positive feedback regulation of microglial glucose metabolism by histone H4 lysine 12 lactylation in Alzheimer’s disease. Cell Metab 34(4):634-648.e636. 10.1016/j.cmet.2022.02.013 35303422 10.1016/j.cmet.2022.02.013

[82] Walker LE, Sills GJ, Jorgensen A, Alapirtti T, Peltola J, Brodie MJ, Marson AG, Vezzani A et al (2022) High-mobility group box 1 as a predictive biomarker for drug-resistant epilepsy: a proof-of-concept study. Epilepsia 63(1):e1–e6. 10.1111/epi.17116 34747496 10.1111/epi.17116

[83] Yang K, Fan M, Wang X, Xu J, Wang Y, Tu F, Gill PS, Ha T et al (2022) Lactate promotes macrophage HMGB1 lactylation, acetylation, and exosomal release in polymicrobial sepsis. Cell Death Differ 29(1):133–146. 10.1038/s41418-021-00841-9 34363018 10.1038/s41418-021-00841-9 PMC8738735

[84] Tretter L, Patocs A (1857) Chinopoulos C (2016) Succinate, an intermediate in metabolism, signal transduction, ROS, hypoxia, and tumorigenesis. Biochim Biophys Acta 8:1086–1101. 10.1016/j.bbabio.2016.03.012 10.1016/j.bbabio.2016.03.012 26971832

[85] Boylston JA, Sun J, Chen Y, Gucek M, Sack MN, Murphy E (2015) Characterization of the cardiac succinylome and its role in ischemia-reperfusion injury. J Mol Cell Cardiol 88:73–81. 10.1016/j.yjmcc.2015.09.005 26388266 10.1016/j.yjmcc.2015.09.005 PMC4780049

[86] Zhang YR, Zhang MD, Zhu W, Yu J, Wang QY, Zhang JJ, Cui YR, Pan XH et al (2020) Succinate accumulation induces mitochondrial reactive oxygen species generation and promotes status epilepticus in the kainic acid rat model. Redox Biol 28:ARTN101365. 10.1016/j.redox.2019.101365 10.1016/j.redox.2019.101365 PMC6854095 31707354

[87] Hasegawa T, Sumita M, Horitani Y, Tamai R, Tanaka K, Komori M, Takenaka S (2014) Gas chromatography-mass spectrometry-based metabolic profiling of cerebrospinal fluid from epileptic dogs. J Vet Med Sci 76(4):517–522. 10.1292/jvms.13-0520 24334864 10.1292/jvms.13-0520 PMC4064135

[88] Roehrs C, Garrido-Sanabria ER, Da Silva AC, Faria LC, Sinhorin VD, Marques RH, Priel MR, Rubin MA et al (2004) Succinate increases neuronal post-synaptic excitatory potentials in vitro and induces convulsive behavior through N-methyl-d-aspartate-mediated mechanisms. Neuroscience 125(4):965–971. 10.1016/j.neuroscience.2004.01.058 15120856 10.1016/j.neuroscience.2004.01.058

[89] Tsortouktzidis D, Schulz H, Hamed M, Vatter H, Surges R, Schoch S, Sander T, Becker AJ et al (2021) Gene expression analysis in epileptic hippocampi reveals a promoter haplotype conferring reduced aldehyde dehydrogenase 5a1 expression and responsiveness. Epilepsia 62(1):e29–e34. 10.1111/epi.16789 33319393 10.1111/epi.16789

[90] Nguyen E, Picklo MJ Sr (2003) Inhibition of succinic semialdehyde dehydrogenase activity by alkenal products of lipid peroxidation. Biochim Biophys Acta 1637(1):107–112. 10.1016/s0925-4439(02)00220-x 12527414 10.1016/s0925-4439(02)00220-x

[91] Pan JW, Kuzniecky RI (2015) Utility of magnetic resonance spectroscopic imaging for human epilepsy. Quant Imaging Med Su 5(2):313–322. 10.3978/j.issn.2223-4292.2015.01.03 10.3978/j.issn.2223-4292.2015.01.03 PMC4379323 25853088

[92] Smeland OB, Hadera MG, McDonald TS, Sonnewald U, Borges K (2013) Brain mitochondrial metabolic dysfunction and glutamate level reduction in the pilocarpine model of temporal lobe epilepsy in mice. J Cereb Blood Flow Metab 33(7):1090–1097. 10.1038/jcbfm.2013.54 23611869 10.1038/jcbfm.2013.54 PMC3705438

[93] Hara Y, Kume S, Kataoka Y, Watanabe N (2021) Changes in TCA cycle and TCA cycle-related metabolites in plasma upon citric acid administration in rats. Heliyon 7(12):ARTN e08501. 10.1016/j.heliyon.2021.e08501 10.1016/j.heliyon.2021.e08501 PMC8654791 34934832

[94] Kovács R, Heinemann U, Steinhäuser C (2012) Mechanisms underlying blood-brain barrier dysfunction in brain pathology and epileptogenesis: role of astroglia. Epilepsia 53:53–59. 10.1111/j.1528-1167.2012.03703.x 23134496 10.1111/j.1528-1167.2012.03703.x

[95] Patel M (2004) Mitochondrial dysfunction and oxidative stress: cause and consequence of epileptic seizures. Free Radical Bio Med 37(12):1951–1962. 10.1016/j.freeradbiomed.2004.08.021 15544915 10.1016/j.freeradbiomed.2004.08.021

