# Supplementary material for: The Metabolic Profile of Plasma During Epileptogenesis in a Rat Model of Lithium–Pilocarpine-Induced Temporal Lobe Epilepsy
Source: Mol Neurobiol. 2025 Feb 4;62(6):7469–83. doi: 10.1007/s12035-025-04719-6 (PMC12078362; doi:10.1007/s12035-025-04719-6)
Supplement: Supplementary file 1 — Supplementary file1 (DOCX 16 KB) [file 12035_2025_4719_MOESM1_ESM.docx]

The metabolic profile of plasma during epileptogenesis in a rat model of lithium–pilocarpine-induced temporal lobe epilepsy

# Fatma Merve Antmen1,2, Emir Matpan^3^, Ekin Dongel Dayanc1,4, Eylem Ozge Savas^5^, Yunus Eken^6^, Dilan Acar^1^, Alara Ak^7^, Begum Ozefe^7^, Damla Sakar^7^, Ufuk Canozer^7^, Sehla Nurefsan Sancak^7^, Ozkan Ozdemir^8^, Osman Ugur Sezerman^9^, Ahmet Tarık Baykal^3,10^, Mustafa Serteser^3,10^, and Guldal Suyen^11,^*

# ^1^Acibadem Mehmet Ali Aydinlar University, Institute of Health Sciences, Department of Physiology, Istanbul, Türkiye

# ^2^Acibadem Mehmet Ali Aydinlar University, Biobank Unit, Istanbul, Türkiye

# ^3^Acibadem Mehmet Ali Aydinlar University, School of Medicine, Department of Medical Biochemistry, Istanbul, Türkiye

# ^4^Acibadem Mehmet Ali Aydinlar University, Vocational School of Health Services, Medical Laboratory Techniques, Istanbul, Türkiye

# ^5^Acibadem Mehmet Ali Aydinlar University, Faculty of Arts and Sciences, Department of Molecular Biology and Genetics, Istanbul, Türkiye

# ^6^Inonu University, Department of Molecular Biology and Genetics, Malatya, Türkiye

# ^7^Acibadem Mehmet Ali Aydinlar University, School of Medicine, Istanbul, Türkiye

# ^8^Acibadem Mehmet Ali Aydinlar University, School of Medicine, Department of Basic Medical Sciences, Medical Biology, Istanbul, Türkiye

# ^9^Acibadem Mehmet Ali Aydinlar University, School of Medicine, Department of Basic Medical Sciences, Biostatistics and Medical Informatics

# ^10^Acibadem Labmed Clinical Laboratories, Istanbul, Türkiye

# ^11^Acibadem Mehmet Ali Aydinlar University, School of Medicine, Department of Physiology, Istanbul, Türkiye

*Correspondence: Guldal Suyen (ORCID: 0000-0003-0863-1547), [guldal.suyen@acibadem.edu.tr](mailto:guldal.suyen@acibadem.edu.tr)

| **Metabolites** | **Fold Change** | **log2(FC)** |
| --- | --- | --- |
| 2-Oxoglutaric acid | 12.622 | 36.579 |
| Acetoacetic acid | 92.824 | 32.145 |
| Dimethylsulfone | 0.2 | -23.219 |
| Creatinine | 0.22222 | -21.699 |
| Acetone | 0.23878 | -20.663 |
| Sarcosine | 0.24444 | -20.324 |
| Creatine | 35.944 | 18.457 |
| 2-Aminobutyric acid | 0.29483 | -17.621 |
| Glycerol | 0.42424 | -1.237 |
| Ethanol | 0.42857 | -12.224 |
| Lysine | 0.42857 | -12.224 |
| Pyruvic acid | 22.105 | 11.444 |
| Threonine | 20.349 | 1.025 |
| Acetic acid | 0.5432 | -0.88043 |
| Lactic acid | 18.209 | 0.86468 |
| Asparagine | 1.08 | 0.848 |
| 2-Hydroxybutyric acid | 1.08 | 0.848 |
| Alanine | 16.074 | 0.68469 |
| Ornithine | 0.63214 | -0.66168 |
| Glycine | 15.355 | 0.6187 |
| 3-Hydroxybutyric acid | 15.074 | 0.59206 |

**Table S1**. The metabolites exhibiting fold-change ≥ 1.5 at 48h post-SE.
