# Supplementary material for: The Metabolic Profile of Plasma During Epileptogenesis in a Rat Model of Lithium–Pilocarpine-Induced Temporal Lobe Epilepsy
Source: Mol Neurobiol. 2025 Feb 4;62(6):7469–83. doi: 10.1007/s12035-025-04719-6 (PMC12078362; doi:10.1007/s12035-025-04719-6)
Supplement: Supplementary file 2 — Supplementary file2 (DOCX 15 KB) [file 12035_2025_4719_MOESM2_ESM.docx]

The metabolic profile of plasma during epileptogenesis in a rat model of lithium–pilocarpine-induced temporal lobe epilepsy

# Fatma Merve Antmen1,2, Emir Matpan^3^, Ekin Dongel Dayanc1,4, Eylem Ozge Savas^5^, Yunus Eken^6^, Dilan Acar^1^, Alara Ak^7^, Begum Ozefe^7^, Damla Sakar^7^, Ufuk Canozer^7^, Sehla Nurefsan Sancak^7^, Ozkan Ozdemir^8^, Osman Ugur Sezerman^9^, Ahmet Tarık Baykal^3,10^, Mustafa Serteser^3,10^, and Guldal Suyen^11,^*

# ^1^Acibadem Mehmet Ali Aydinlar University, Institute of Health Sciences, Department of Physiology, Istanbul, Türkiye

# ^2^Acibadem Mehmet Ali Aydinlar University, Biobank Unit, Istanbul, Türkiye

# ^3^Acibadem Mehmet Ali Aydinlar University, School of Medicine, Department of Medical Biochemistry, Istanbul, Türkiye

# ^4^Acibadem Mehmet Ali Aydinlar University, Vocational School of Health Services, Medical Laboratory Techniques, Istanbul, Türkiye

# ^5^Acibadem Mehmet Ali Aydinlar University, Faculty of Arts and Sciences, Department of Molecular Biology and Genetics, Istanbul, Türkiye

# ^6^Inonu University, Department of Molecular Biology and Genetics, Malatya, Türkiye

# ^7^Acibadem Mehmet Ali Aydinlar University, School of Medicine, Istanbul, Türkiye

# ^8^Acibadem Mehmet Ali Aydinlar University, School of Medicine, Department of Basic Medical Sciences, Medical Biology, Istanbul, Türkiye

# ^9^Acibadem Mehmet Ali Aydinlar University, School of Medicine, Department of Basic Medical Sciences, Biostatistics and Medical Informatics

# ^10^Acibadem Labmed Clinical Laboratories, Istanbul, Türkiye

# ^11^Acibadem Mehmet Ali Aydinlar University, School of Medicine, Department of Physiology, Istanbul, Türkiye

# *Correspondence: Guldal Suyen (ORCID: 0000-0003-0863-1547), [guldal.suyen@acibadem.edu.tr](mailto:guldal.suyen@acibadem.edu.tr)

| **Metabolites** | **Fold Change** | **log2(FC)** |
| --- | --- | --- |
| 2-Hydroxybutyric acid | 0.16903 | -25.647 |
| Proline | 44.318 | 21.479 |
| Glycerol | 0.40878 | -12.906 |
| Ornithine | 24.182 | 12.739 |
| 2-Aminobutyric acid | 21.691 | 11.171 |
| Choline | 21.356 | 10.946 |
| 2-Oxoglutaric acid | 0.52835 | -0.92043 |
| Pyruvic acid | 18.798 | 0.91059 |
| Acetone | 18.273 | 0.86969 |
| Ethanol | 1.08 | 0.848 |
| Asparagine | 1.08 | 0.848 |
| 3-Hydroxybutyric acid | 1.08 | 0.84436 |
| Lysine | 0.63636 | -0.65208 |
| Glutamic acid | 0.64665 | -0.62893 |

**Table S2.** The metabolites exhibiting fold-change ≥ 1.5 at 1wk post-SE.
