# Supplementary material for: The Metabolic Profile of Plasma During Epileptogenesis in a Rat Model of Lithium–Pilocarpine-Induced Temporal Lobe Epilepsy
Source: Mol Neurobiol. 2025 Feb 4;62(6):7469–83. doi: 10.1007/s12035-025-04719-6 (PMC12078362; doi:10.1007/s12035-025-04719-6)
Supplement: Supplementary file 3 — Supplementary file3 (DOCX 15 KB) [file 12035_2025_4719_MOESM3_ESM.docx]

The metabolic profile of plasma during epileptogenesis in a rat model of lithium–pilocarpine-induced temporal lobe epilepsy

# Fatma Merve Antmen1,2, Emir Matpan^3^, Ekin Dongel Dayanc1,4, Eylem Ozge Savas^5^, Yunus Eken^6^, Dilan Acar^1^, Alara Ak^7^, Begum Ozefe^7^, Damla Sakar^7^, Ufuk Canozer^7^, Sehla Nurefsan Sancak^7^, Ozkan Ozdemir^8^, Osman Ugur Sezerman^9^, Ahmet Tarık Baykal^3,10^, Mustafa Serteser^3,10^, and Guldal Suyen^11,^*

# ^1^Acibadem Mehmet Ali Aydinlar University, Institute of Health Sciences, Department of Physiology, Istanbul, Türkiye

# ^2^Acibadem Mehmet Ali Aydinlar University, Biobank Unit, Istanbul, Türkiye

# ^3^Acibadem Mehmet Ali Aydinlar University, School of Medicine, Department of Medical Biochemistry, Istanbul, Türkiye

# ^4^Acibadem Mehmet Ali Aydinlar University, Vocational School of Health Services, Medical Laboratory Techniques, Istanbul, Türkiye

# ^5^Acibadem Mehmet Ali Aydinlar University, Faculty of Arts and Sciences, Department of Molecular Biology and Genetics, Istanbul, Türkiye

# ^6^Inonu University, Department of Molecular Biology and Genetics, Malatya, Türkiye

# ^7^Acibadem Mehmet Ali Aydinlar University, School of Medicine, Istanbul, Türkiye

# ^8^Acibadem Mehmet Ali Aydinlar University, School of Medicine, Department of Basic Medical Sciences, Medical Biology, Istanbul, Türkiye

# ^9^Acibadem Mehmet Ali Aydinlar University, School of Medicine, Department of Basic Medical Sciences, Biostatistics and Medical Informatics

# ^10^Acibadem Labmed Clinical Laboratories, Istanbul, Türkiye

# ^11^Acibadem Mehmet Ali Aydinlar University, School of Medicine, Department of Physiology, Istanbul, Türkiye

*Correspondence: Guldal Suyen (ORCID: 0000-0003-0863-1547), [guldal.suyen@acibadem.edu.tr](mailto:guldal.suyen@acibadem.edu.tr)

| **Metabolites** | **Fold Change** | **log2(FC)** |
| --- | --- | --- |
| Acetoacetic acid | 0.21086 | -22.456 |
| Acetone | 0.22751 | -2.136 |
| Succinic acid | 24.292 | 12.805 |
| Ornithine | 20.893 | 1.063 |
| Pyruvic acid | 19.929 | 0.99484 |
| Glycerol | 1.787 | 0.83751 |
| Lactic acid | 16.925 | 0.75916 |
| Creatinine | 0.6 | -0.73697 |

**Table S3**. The metabolites exhibiting fold-change ≥ 1.5 at 6wk post-SE.
